# Macaronesia Acts as a Museum of Genetic Diversity of Relict Ferns: The Case of *Diplazium caudatum* (Athyriaceae)

**DOI:** 10.3390/plants10112425

**Published:** 2021-11-10

**Authors:** Samira Ben-Menni Schuler, Jesús Picazo-Aragonés, Fred J. Rumsey, Ana Teresa Romero-García, Víctor N. Suárez-Santiago

**Affiliations:** 1 Department of Botany, Faculty of Sciences, University of Granada, 18071 Granada, Spain; samira@ugr.es (S.B.-M.S.); jpicazo@us.es (J.P.-A.); atromero@ugr.es (A.T.R.-G.); 2 Department of Life Sciences, Natural History Museum, Cromwell Road, London SW7 5BD, UK; f.rumsey@nhm.ac.uk

**Keywords:** fern phylogeography, genetic diversity, Macaronesia, mating systems, microsatellites, plastid DNA, refugia, species distribution modelling, Tertiary relicts

## Abstract

Macaronesia has been considered a refuge region of the formerly widespread subtropical lauroid flora that lived in Southern Europe during the Tertiary. The study of relict angiosperms has shown that Macaronesian relict taxa preserve genetic variation and revealed general patterns of colonization and dispersal. However, information on the conservation of genetic diversity and range dynamics rapidly diminishes when referring to pteridophytes, despite their dominance of the herbaceous stratum in the European tropical palaeoflora. Here we aim to elucidate the pattern of genetic diversity and phylogeography of *Diplazium caudatum*, a hypothesized species of the Tertiary Palaeotropical flora and currently with its populations restricted across Macaronesia and disjunctly in the Sierras de Algeciras (Andalusia, southern Iberian Peninsula). We analysed 12 populations across the species range using eight microsatellite loci, sequences of a region of plastid DNA, and carry out species-distribution modelling analyses. Our dating results confirm the Tertiary origin of this species. The Macaronesian archipelagos served as a refuge during at least the Quaternary glacial cycles, where populations of *D. caudatum* preserved higher levels of genetic variation than mainland populations. Our data suggest the disappearance of the species in the continent and the subsequent recolonization from Macaronesia. The results of the AMOVA analysis and the indices of clonal diversity and linkage disequilibrium suggest that *D. caudatum* is a species in which inter-gametophytic outcrossing predominates, and that in the Andalusian populations there was a shift in mating system toward increased inbreeding and/or clonality. The model that best explains the genetic diversity distribution pattern observed in Macaronesia is, the initial and recurrent colonization between islands and archipelagos and the relatively recent diversification of restricted area lineages, probably due to the decrease of favorable habitats and competition with lineages previously established. This study extends to ferns the concept of Macaronesia archipelagos as refugia for genetic variation.

## 1. Introduction

Macaronesia, which comprises the mid-Atlantic oceanic archipelagos of the Azores, Canaries, Cape Verde, Madeira and Selvagems, is considered an exceptional biogeographical and evolutionary model system [1]. Ref. [2] proposed the relictualism hypothesis for the Macaronesian flora, since the Macaronesian endemic element was a relict of the formerly widespread subtropical lauroid flora that lived in Southern Europe during the Tertiary, especially during the Palaeogene Period (66–23.03 Ma; [3,4]). A growing amount of evidence, mainly from population genetics and phylogeographic studies of endemic angiosperms, supports the Macaronesian archipelagos acted as efficient refugia for genetic diversity of relict taxa [5,6,7,8,9,10]. In addition, has been revealed general patterns of colonization and dispersal, including single colonization of a common ancestor followed by rapid radiation, multiple independent colonizations, back-colonization to the continent from Macaronesia, and a predominantly Macaronesian–Western Mediterranean source area for the endemic lineages [11]. Unlike angiosperms, cryptogams have been much less studied, despite the fact that the region is considered a globally important centre of pteridophyte and bryophyte diversity [12,13], and the possibility is that from their study new evidence is obtained on the processes that underlie the evolution of island plant diversity [14].

Ref. [15], in their phylogenetic study of floristic data, found the pteridophyte flora of the Macaronesian archipelagos (except Cape Verde) being monophyletic and related to the European pteridophytes, which is consistent with the relictualism hypothesis for the Macaronesian flora. Numerous ferns are considered lineages of Tertiary origin, especially most of the Mediterranean region [16,17], which were the main component of the herbaceous layer of the European lauroid forest [18,19,20]. The climate deterioration during the Tertiary, especially from the mid-Miocene onwards, together with Pleistocene glaciations, caused the decline of this flora, whose representatives survived in refugia present along the European Atlantic coast and Macaronesia [15,21,22,23,24,25,26]. The hypothesis of relictualism has been tested recently for 18 representative angiosperms of the Macaronesian laurel forest, using fossil data, ancestral area reconstruction, and molecular dating [27]. These authors suggested that most taxa were not Tertiary relicts but rather Plio–Pleistocene in origin [27]. In pteridophytes, the only studies that have tested this hypothesis are those conducted by us in *Vandenboschia speciosa* (Willd.) G. Kunkel and *Culcita macrocarpa* C. Presl [28]. The origin of these species dates back to the Middle Miocene and Early Miocene, respectively, with *V. speciosa* reaching Macaronesia during the Pliocene-Pleistocene (the low resolution of the plastid sequences avoided identifying and dating this event for *C. macrocarpa*).

Phylogeographic analyses in Macaronesian mosses and liverworts show the presence of gene flow and several colonization events both among archipelagos and between those and continents [10,29,30]. However, as far as pteridophytes are concerned, there are very few phylogeographic studies and only four (as far as we know), in addition to the two mentioned above carried out by us [28], that include Macaronesian populations: one focused on the Macaronesian *Asplenium hemionitis* L. [31], a preliminary study of *Vandenboschia speciosa* from Macaronesia and continental Europe [32], a comparison of Macaronesian examples of the *Polypodium macaronesicum* A. E. Bobrov complex with the continental *P. cambricum* L. [33], and another that included two Canarian populations of *Asplenium ceterach* L. in a large-scale European study [34]. Contrary to expectations according to their dispersive capacity, and especially in European pteridophytes of supposedly Tertiary origin, the phylogeographic studies resulted in strong genetic structuring [33,34,35,36,37,38]. The shortage of studies on Macaronesian ferns prevents inferring general phylogeographic patterns and colonization stories, but these few studies, together with the population genetics study by [36], show the existence of population genetic structure between archipelagos, different genetic relationships among archipelagos and between archipelagos and the mainland, phenomena of multiple colonization, and evidence of back-colonization to the continent [28,31,32,36].

Pteridophytes, including lycophytes and monilophytes, are organisms with high dispersive capacity [39], by spores, which favors population connectivity and decreased genetic structure [40]. This may account for its low levels of endemism by comparison with angiosperms, as occur in Macaronesia. The Madeiran pteridophyte flora exhibits the highest levels of endemism of the region (9.7%) and the Canaries the lowest (5.5%), a diversity pattern markedly different to that of angiosperms (lowest: 22.5% in Madeira, highest: 45.5 Canaries; [14]). However, apart from the dispersive capacity, other biological peculiarities of pteridophytes influence the colonization process and the genetic diversity of their populations, such as the scarcity and the disjunct distribution of suitable habitats with high environmental humidity and warm temperatures. The disjunction of this type of environment represents a significant barrier to populations dispersion and connectivity, and explains the strong genetic structure across a wide geographical scale observed in many studies; despite the high dispersive capacity of ferns [34,35,36,37,38,41,42]. The fertilization is always a post-dispersal process (it occurs on the gametophyte originated from spores) and then the probability of colonization of a new habitat will depend on the breeding system of the species. In homosporous ferns, three mating systems can occur: intra-gametophytic selfing, in which a sporophyte is produced from a single bisexual gametophyte; inter-gametophytic selfing, in which fertilization occurs between two gametophytes from the same sporophyte; and inter-gametophytic outcrossing, where the intervening gametophytes come from different sporophytes. Colonization of a new habitat will be more probable for ferns with intra-gametophytic selfing, favouring the population establishment even from single or few spores [43,44,45,46,47]. Moreover, many ferns have ability to propagate vegetatively. All these peculiarities strongly influence the modelling of the structuring of the taxa’s genetic diversity [47,48,49]. In brief, intra-gametophytic selfing, like vegetative propagation, would lead to populations with completely homozygous individuals and low levels of genetic diversity, and strongly differentiate from each other; polyploidy has been postulated as a mechanism for storing genetic variation in these populations [50]. Inter-gametophytic selfing, in which the spores generating the gametophytes have undergone meiotic segregation in the sporophyte, will result in higher levels of genetic diversity than intra-gametophytic selfing but will be relatively low with respect to individuals resulting from inter-gametophytic outcrossing. Outcrosser fern populations will be those with the highest levels of genetic diversity and less structured.

In the present study, we investigate the phylogeography of *Diplazium caudatum* (Cav.) Jermy (Athyriaceae), a supposed outcrosser fern distributed across Macaronesia which also occurs disjunctly in the Sierras de Algeciras (Cádiz, southern Iberian Peninsula). Chromosome counts carried out by [51], on Madeira populations, and by [52], on Azores populations, indicate that this is a diploid species (2*n* = 82). Along with many other Macaronesian pteridophytes, *D. caudatum* has been supposed to be a Boreotropical remnant resulting from the decrease in temperature at the end of the Tertiary, with subsequent colonisation of the Atlantic islands [15,20,53]. During the end of the Neogene and the Pleistocene glaciations, it is believed that the continental populations of those species became restricted to refugial pockets (the so-called Iberian refugia: Algeciras Mountains, Algarve, Galicia, Cantabrian Cornice) but the Macaronesian populations remained unaffected due to the buffering effect of the Atlantic Ocean [1,15]. Therefore, we could expect the gene flow between the Macaronesian archipelagos and the mainland was restricted, thus the different regions became genetically divergent due to isolation and independent evolution. In this paper we test this hypothesis using species-specific microsatellites, one plastid marker (ptDNA), and species distribution modelling (SDM), and fulfilling the following tasks: (a) to infer when the species originates to test for the Tertiary relict hypothesis; (b) to determine the levels of genetic diversity and its structuring at the scale of the total distribution range of the species; (c) to estimate the importance of mating systems on population composition and to evaluate the effect of clonality on its intraspecific genetic structure; (d) to establish the evolutionary relationships between the different populations/genetic lineages of *D. caudatum* and to know the geological-climatic events and the population processes that explain their distribution; and (e) to know how climate change could affect the distribution of this species in the future.

## 2. Material and Methods

### 2.1. Plant Material

**Figure 1:**
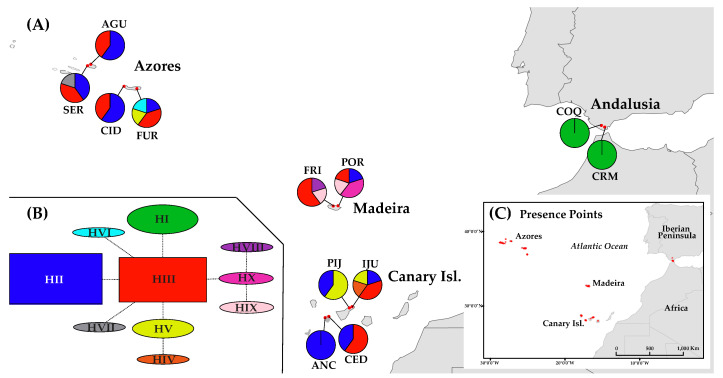
Distribution and haplotype composition of the population sampled of *D. caudatum*. (**A**) Geographical distribution of haplotypes (see Table 1 for population code); pie charts indicate haplotype frequency. (**B**) Network representing the relationships between haplotypes of the ptDNA; inferred following the statistical parsimony method, with TCS. (**C**) Location of presence records (black dots) used for species distribution modelling (SDM).

Samples of *D. caudatum* were obtained from 12 populations in four geographical regions across its distribution range: Andalusia, Azores, Canary Islands and Madeira. We were unable to sample the known population in Sto. Antão island of Cape Verde for logistical reasons. The number of populations per region was between two and four and the number of sampled individuals per population varied from 17 to 30 (335 in total; Table 1; Figure 1).

### 2.2. DNA Extraction, Microsatellite Genotyping, and ptDNA Sequencing

Total genomic DNA of the 335 individuals was extracted from silica dried fronds following a modified low-salt CTAB extraction protocol [54]. All individuals were genotyped for eight recently developed microsatellite loci (see Table S1 for microsatellite characteristics). Genotyping was performed on an ABI PRISM^®^ 3100-Avant Genetic Analyzer (Applied Biosystems, Foster City, CA, USA). Alleles were scored using GENEMARKER v1.85 (SoftGenetics, State College, PA, USA).

For ptDNA analysis, a subsample of 60 individuals (five per population) was used. The plastid marker used was the intergenic spacer *trn*S-*trn*G, the most polymorphic region of the four tested (*trn*S-*trn*G, *trn*H-*psb*A, *trn*L-*trn*F and *rpL*32-*trn*L), and a partial sequence (toward 5′ end) was amplified and sequenced. We could not use the entire region due to the presence of long mononucleotide runs, from about 16 base pairs (bp), which prevented sequencing. The specific primers DC-trnS-GCU2: 5′-ATTAGCAATCCGACGCTTTA–3′ and DC-trnG-UCC2: 5′-ATTCGAACCCGCATCAGTAG-3′, designed from the general ones [55], were used to amplify the entire *trn*S-*trn*G region in all individuals. Then, the internal primer 5′-trnG2S [55] was used to sequence the 5′ fragment. PCR reactions were performed in 25-µL reactions containing 50 ng of genomic DNA, 1.25 µM of each primer, and 12.5 µL of the Kapa 2G Robust HotStart ReadyMix (Kapa Biosystems, MA, USA). Cycling parameters were those described in [55]. Sequencing was performed on an ABI PRISM^®^ 3100-Avant Genetic Analyzer (Applied Biosystems, Foster City, CA, USA). The resulting sequences were aligned by eye in the alignment editor BIOEDIT v7.0.5.3 [56].

### 2.3. Clonality and Genetic Diversity

#### 2.3.1. Microsatellites

In order to infer the clonal identity of the sampling units (all individuals sampled), first we tested the resolutive power of the eight microsatellite markers by estimating the genotype accumulation curve using the function *genotype_curve* from the R package POPPR v2.8.3 [57]. This function samples, using a Monte Carlo procedure, random subsets of loci and examine the robustness of the inferred clonal memberships. Then, we used MLGsim v2.0 [58] for calculating the probability that repeated multilocus genotypes (MLGs) originate from different sexual reproduction events (*p*_sex_; being different genets), based on the observed allele frequencies and the sample size of the data set, and taking into account departures from Hardy–Weinberg equilibrium (HWE) when estimating genotypic probabilities (*p*_gen_ (*F*_IS_)), for a more conservative estimate of *p*_sex_; [59]. The significance values of *p*_sex_ were obtained by comparison with the distribution of 1000 simulated *p*_sex_ values. Finally, to define the clonal lineages or multiple locus lineages (MLLs; i.e., different MLGs belonging to a distinct or the same clone) we analysed the distribution of the frequencies of genetic distances between pairs of MLGs, with the function *mlg.filter* and using Bruvo distances on POPPR. The genetic threshold distance under which two MLGs were considered the same MLL was estimated using the farthest neighbour method.

The clonality descriptors were calculated with the function *poppr* on POPPR as follows: (a) to characterize the clonal richness, we calculated: number of MLLs, number of expected MLLs (eMLLs), and clonal richness (R) corrected for sampling size; (b) to characterize the genotype diversity, we calculated: the Simpson’s index (lambda; corrected for sampling size), and the clonal evenness index (E.5), which shows how equally each MLL is represented. Finally, we calculated the standardized association index (*r*_d_; [60]) to test the predominant reproductive model (sexual, where linkage among loci is not expected, vs. clonal, where significant disequilibrium is expected due to linkage among loci). This latter index was also estimated applying correction for clones, and then using only one individual per MLL, in order to test the effect of partial clonality. The significance of *r*_d_ was tested with a permutation test (10,000 permutations). All descriptors were estimated both at population and geographical region levels.

To calculate genetic diversity descriptors we used all sampling units, following the recommendation of [61] and because we consider that this way the real genetic structure of *D. caudatum* populations is better represented. Thus, we calculated: the number of alleles (*A*) and allelic richness (*Ar*), rarefacting to the smallest sample size, using the R package HIERFSTAT v0.04-30 [62] with the functions *allele.count* and *allelic.richness*, respectively; the observed and expected heterocigosity (*H*_O_ and *H*_E_, respectively), and fixation index *F*_IS_ and HWE using GENODIVE v3.0 [63]. Furthermore, in order to test the effect of asexual reproduction on intrapopulation genetic diversity, we also calculated genetic descriptors (*H*_O_, *H*_E_ and *F*_IS_) using only one individual per MLL per population, and a permutation test using the Osx-statistic [64] was performed to explore significant differences between data sets (including vs. excluding clones) with GENODIVE. All genetic descriptors were calculated both at population and geographical region levels.

#### 2.3.2. ptDNA

Genetic diversity was assessed by the number of haplotypes (*ha*), haplotype diversity (*Hd*), and nucleotide diversity (*π*) calculated using ARLEQUIN v3.5.2.2 [65]. All diversity indices were calculated both at population and geographical region levels.

### 2.4. Genetic Structure and Phylogeography

Standard and hierarchical analyses of molecular variance (AMOVA; [66]) were used to test the partitioning of genetic variability within samples, within and between populations, and between the four geographical regions. For microsatellites, these analyses were made with all sampling units and with only one individual per MLL per population, using the function *poppr.amova* on POPPR and the function *randtest*, running 1000 replicates, to test for significance. For ptDNA we used the program ARLEQUIN, and the significance was tested with 10,000 permutations. Population genetic structure was analysed using different approaches with our microsatellite data. First, pairwise *F*_ST_ values were calculated, with both all sampling units and with only one individual per MLL per population, between populations using GENODIVE; the significance of *F*_ST_ was tested by a permutation test with 10,000 permutations. We compared the values obtained with and without clones using the Spearman’s correlation coefficient. Second, the Bayesian algorithm implemented in STRUCTURE v2.3.4 [67] was used to evaluate the number of genetic clusters (*K*) with all sampling units. The number of clusters tested ranged from one to 13, with ten replicates per *K*, using the no admixture model and independent allele frequencies. The burn-in period and Markov Chain Monte Carlo (MCMC) iterations were set to 50,000 and 10^6^, respectively. The optimal number of clusters was estimated with the online tool STRUCTURESELECTOR [68]. We identified the uppermost hierarchical level of genetic structure using the delta *K*-method (Δ*K*; [69]). To explore other levels of genetic partitioning, we used the four independent estimators proposed by [70] (MedMedK, MedMeanK, MaxMedK, and MaxMeanK) considering a membership coefficient threshold of 0.5. To align and visualize the STRUCTURE output across the 10 replicates, we used the online tool CLUMPAK [71]. Third, the genetic structure was also assessed using a model-free multivariate statistics-based clustering method, a discriminant analysis of principal components (DAPC) on R package ADEGENET [72] using all sampling units. The function *xvalDapc* from ADEGENET was used to select by cross-validation the correct number of principal components with 1000 replicates using a training set of 90% of the data. The number of principal components was chosen based on the criteria that it had to produce the highest average percentage of successful reassignment and lowest root mean squared error [72].

In order to explore the evolutionary relationships and geographical distribution of ptDNA haplotypes, a haplotype network was reconstructed following the statistical parsimony method [73] as implemented in TCS v1.21 [74].

### 2.5. Gene Flow and Demographic Analyses

We tested the connectivity among populations by estimating the migration rates among them, with all sampling units, using microsatellite data. Thus, to know whether there was recent (over two to three generations) gene flow between the populations, we estimated migration rates (*m*) between all individual populations using a Bayesian assignment test with the software BAYESASS v1.3 [75]. As program settings, the default values were used (MCMC iterations, 3 × 10^6^; length of the burn-in, 999,999; sampling frequency, 2000; delta value, 0.15). Isolation by distance (IBD) was tested for the 12 populations using regression of pairwise *F*_ST_ distances (determined with GENODIVE using them transformed as *F*_ST_/(1 − *F*_ST_)) and logarithms of geographical distances between populations, using a Mantel test in GENODIVE. Fu’s *F* [76] and Tajima’s *D* [77] neutrality tests were carried out with ptDNA, to detect possible historical demographic processes (expansion or contraction), using ARLEQUIN. Both tests were performed considering populations and geographical regions. The level of significance of both statistics was obtained by 1000 simulated samples. In addition, ptDNA sequences were used to test for evidence of population size fluctuations between *D. caudatum* regions by constructing Bayesian Skyline Plots with BEAST (BSP; [78]).

### 2.6. Haplotype Phylogeny and Dating

Phylogenetic relationships among ptDNA haplotypes of *D. caudatum* and the outgroup species were inferred using Bayesian Inference (BI), with MrBayes v3.1.2 [79]. As outgroup species, we use the sequences obtained by [80] of *D. bellum*, *D. dilatatum*, *D. dushanense*, *D. striatum*, and *D. unilobum* (accession numbers: KY427343-KY427347). The analysis was run using the selected substitution model, Hasegawa-Kishino-Yano, with proportion of invariable sites (HKY + I; [81]) determined by jModeltest v2.1.10 [82], and included two million generations with two simultaneous runs (Markov Chain Monte Carlo, MCMC), starting from random trees that were sampled every 100 generations. The TRACER v1.7 program [83] was used to visualize the results and confirm the convergence and stationarity of the races. 25% of the initial trees, from the pre-convergent phase (burn-in phase), were removed. The rest of the trees were used to construct the phylogenetic consensus tree (“50% majority rule consensus”).

To date divergence events between plastidial haplotypes we used BEAST v2.0 computer program [84]. The divergence times were obtained using the selected HKY + I substitution model, a strict molecular clock, tested with PAML v4 [85], and a background tree (“priors”) according to the “Calibrated Yule Model”. The nodes of the tree were constricted according to the phylogeny obtained with MrBayes and calibrated using a normal distribution, establishing the confidence interval, for the estimated divergence time for *D. caudatum* according to [53]. The length of the Markov chains was established at 10 million generations, sampling the trees every 1000 generations. All these parameters were established with the BEAUti program included in BEAST. TRACER v1.7 was used to visualize the results obtained and confirm the convergence of the chains and eliminate the sampled trees during the pre-convergent phase (“burn-in”). The initial 10% of trees sampled in each race were eliminated. The trees obtained were joined in a tree of maximum credibility of the clade (“maximum clade credibility tree”) using TREEANNOTATOR, also included in BEAST, and was visualized using the tree editor FIGTREE v1.4.3 (http://tree.bio.ed.ac.uk/software/figtree/, accessed 15 July 2017).

### 2.7. Species Distribution Modelling

To identify potential refugial and future distribution areas for *D. caudatum*, species distribution modelling (SDM) was performed. This analysis requires presence data of the studying species and environmental variables. As environmental data, we used 19 BIOCLIM variables at a resolution of 2.5 arc-minutes (ca. 5 km) representing different time periods during past, present and future climatic conditions. Past and current climate data were available from the WorldClim database (www.worldclim.org, accessed 15 July 2017; [86]) and included data for the current-day period (1950–2000), the Last Glacial Maximum (LGM; *c*. 21 ka) simulated by CCSM model (the Community Climate System Model), and for the Last Interglacial period (LIG; *c*. 120ka). We obtained predictions for future climatic conditions in 2080 for the most impacting IPCC’s climate scenario: RCP 8.5 [87] available through the CCAFS Climate portal (www.ccafs-climate.org, accessed 15 July 2017). Soil data were obtained from SoilGrids.org [88] but were not used with past climatic conditions because of the lack of this type of maps. Highly correlated variables (Pearson’s R ≥ 0.8) were reduced to eight uncorrelated variables (Table S2) used as predictors to calibrate the distribution models. Species occurrence data were obtained from a collection of references in databases (the Global Biodiversity Information Facility data portal (http://www.gbif.org/; accessed 15 July 2017), the Biodiversity databank of the Canary Islands (http://www.biodiversidadcanarias.es/atlantis/common/index.jsf; accessed 16 June 2013), and the Azores Biodiversity databank (http://www.atlantis.angra.uac.pt/atlantis/common/index.jsf; accessed 1 February 2014), literature [52,89], and our own field records. A total of 323 presence records were finally compiled (Figure 1C). To perform the SDM we applied Maximum Entropy Modelling implemented in the software package MAXENT 3.4.1 [90]. Models were generated using cross-validation of five replicate runs. Model performance was assessed based on the area under the receiver operating characteristic curve (AUC). The contribution of each predictor variable in the model was analysed by the permutation importance and percent contribution coefficients (Table S2). A final reduced model including the most important variables (Mean Diurnal Range and Minimum Temperature of Coldest Month), was finally computed [91].

## 3. Results

### 3.1. Clonality and Genetic Diversity

#### 3.1.1. Microsatellites

A total of 294 MLGs were detected between the 335 sampling units. In relation to the resolutive power of the eight microsatellites used in this study, with seven loci almost 100% of the MLGs of *D. caudatum* are resolved as shown in the genotypic accumulation curve (Figure S1). The genetic threshold distance, under which two MLGs were considered to belong to the same MLL, was 0.039 (Figure S2). The total number of MLLs was 282 distributed among 294 individuals (genets) across the populations, with a different number of clones between populations and geographical regions (Table 2; Figure S3a,b). However, eight MLLs were distributed among individuals of different populations and, therefore, we kept these individuals with their original MLGs resulting finally in 290 MLLs (Table 2). Between two and thirty MLLs were detected across all the populations. Canary Islands was the region that retained the highest number of MLLs (114) and Madeira showed the highest clonal richness (R = 0.983). In general, all Macaronesian populations and archipelagos showed similar and high values of clonal richness and genotype diversity; while Andalusia was the region with the greatest clonal prevalence, with few MLLs per population, and only some that were dominant (Table 2; Figure S3a,b). The association index was significant, indicating linkage disequilibrium, for Andalusia and the Canary Islands, at the regional level, and for COQ, CED and FUR, at the population level (Table 2). Only for COQ the association index was not significant when one individual per MLL per population was considered.

In total, 101 alleles were observed from the eight loci surveyed (mean = 12.6). All geographical regions and populations, except CRM (with only one polymorphic locus; Andalusia) and CID (Azores), deviated from HWE when all individuals were included and considering only one individual per MLL per population. No genetic diversity index changed significantly when only one individual per MLL per population was considered (OSx_*H*_O__ = 0.01; *p* = 0.83; OSx_*H*_E__ = 0.02; *p* = 0.74; OSx_*F*_IS__ = 0.007; *p* = 0.91). At the population and regional level, it was Madeira which showed the highest diversity values and Andalusia the lowest; the Azores and the Canary Islands showed similar diversity values (Table 3).

#### 3.1.2. ptDNA

Plastid DNA sequence alignment included 60 sequences in total, with 722 base pairs (bp) in length and it included nine variable positions. The total number of haplotypes found was 10. Results for the diversity indices are shown in Table 3. At the population level, the mean diversity values for the ptDNA were *Hd* = 0.784 and *π* = 0.00176, and both the Andalusian populations and ANC (Azores) showed null diversity values. At the regional level, the most diverse region was Madeira and the least diverse, Andalusia (Table 3).

### 3.2. Genetic Structure and Phylogeography

AMOVA analyses, both standard and hierarchical, using microsatellites showed that the highest proportion of diversity is always found within samples and that the proportion of diversity and differentiation between populations or between regions, when considered, although significant was always much lower. The same results were obtained when only one individual was considered per MLL per population (Table 4). Plastid AMOVA results showed that the highest percentage of genetic variation resides within populations (64.41%, *p* < 0.001), and that the 29.44% (*p* < 0.001) was at the inter-regional level. The variation found between population within geographical regions was low and not significant (Table 4).

Pairwise *F*_ST_ values with and without clonal individuals showed a significant correlation (*r* = 0.992; *p* = 0.0001); lower and less paired significant differences were found when clones were excluded (Table S3). Considering all sampling units, all pairwise comparisons were significant less FUR-SER (Azores). At an intra-regional level, Azores and Madeira showed low population differentiation; however, in the Canary Islands, the intra-regional values were similar (even higher for comparisons involving ANC population) to the inter-regional ones in the comparisons with Azorean and Madeiran populations. In Andalusia, the intra-regional *F*_ST_ value was high, while at the inter-regional level both Andalusian populations were the most differentiated, especially CRM. The latter, however, showed no significant values in all comparisons when only one individual was considered per MLL per population (Table S3).

**Figure 2:**
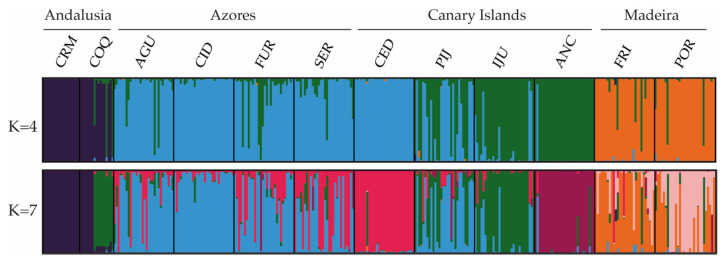
Estimated genetic structure based on microsatellite data using the Bayesian approach implemented in STRUCTURE. Histograms of individual assignment to clusters show the two most probable structuring, *K* = 4 and *K* = 7.

The Bayesian clustering method, as implemented in STRUCTURE, recognized four genetic clusters (*K* = 4; Figure 2) as the uppermost hierarchical level of genetic partitioning according to the highest Δ*K* peak (Figure 2). In general, individuals from the populations of Andalusia, Azores and Madeira could be identified as belonging to three distinct clusters (CL1, CL2 and CL3; respectively). The fourth cluster (CL4) was represented mainly by individuals from the Canarian populations ANC and IJU. Of the other two Canarian populations, all CED individuals belonged to the CL3 cluster, while in PIJ we found a mixture of individuals of the CL3 and CL4 clusters. The CL4 cluster was also represented in three populations of Azores and in the Andalusian COQ by a small proportion of individuals. The results interpreted using the method of [70] revealed seven clusters (*K* = 7; Figure 2) as the most likely substructure. In this case, ANC, CED, and POR appeared differentiated, being assigned in their own cluster. In addition, the relationship between COQ and IJU, and between the Azorean and CED populations, was more evident.

**Figure 3:**
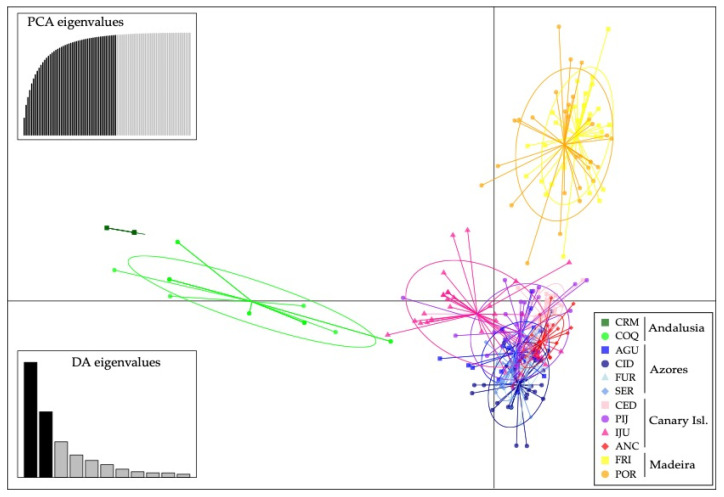
Result of the discriminant analysis of principal components (DAPC) using microsatellites.

Discriminant analysis of principal component yielded in a similar result as with STRUCTURE for *K* = 4 (Figure 3).

The representation on a map of the ptDNA haplotype frequencies and distributions suggests a slight geographical structuring of them (Figure 1A). While haplotypes H-II and H-III are the most frequent and widespread, being present in all the sampled Macaronesian archipelagos, H-I is exclusive from Andalusia, H-V is present in the Azores and the Canary Islands, H-IV is exclusive from the Canary Islands, H-VI and H-VII from the Azores and H-VIII, H-IX and H-X from Madeira (Figure 1A, Table 3). The ptDNA network shows H-II and H-III as the most frequent haplotypes (Figure 1B), H-III occupying the central position from which most haplotypes differ by a mutation. H-IV from the Canary Islands is related to H-V, from which differs in one change, and the three private haplotypes from Madeira are closely related, with H-VIII and H-IX differing each from H-X by a mutation (Figure 1B).

### 3.3. Gene Flow and Demographic Analyses

The results of BAYESASS indicated that current exchange of genes could be occurring from AGU to the other Azorean populations (CID, FUR, SER), and to the Canarian PIJ and IJU (Table S4), since the estimates of *m* obtained exceeded the threshold value of 0.110 (upper value of the confidence interval when there is no information in the data). The Mantel test showed the existence of isolation by distance across the populations (*r* = 0.217, *p* < 0.01); however, when we excluded populations from Andalusia the test was not significant (*r* = 0.093, *p* = 0.179).

**Figure 4:**
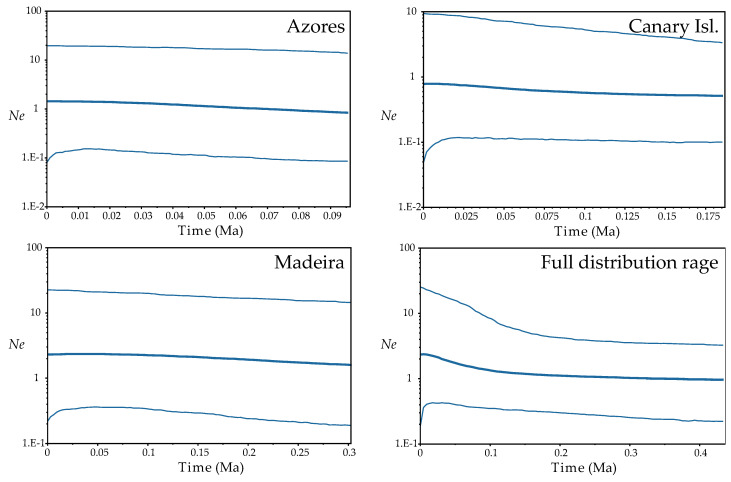
Demographic analyses (Bayesian Skyline Plots) based on coalescence considering each Macaronesian archipelago separately and the full distribution range of the species, inferred from ptDNA and implemented with BEAST. Plots depict changes in effective population size (*N*_e_) as a function of time (in million years ago, Ma); in each plot, the centre line is the median estimate, and the upper and lower lines delimit the highest posterior density (HPD) 95% confidence intervals for *N*_e_.

Fu’s *F* and Tajima’s *D* tests resulted not significative except for the *F* in FUR, IJU and POR populations, being negative and then indicating expansion of these populations (Table S5). At the regional level, although not significant, the trend of the results of these tests suggested population expansion from the Azores and Madeira (highly negative values). The BSP analyses found evidence for range expansion when we considered all the distribution range of the species, this starting from around 100 thousand years ago (Ka; beginning of the Würm glaciation). No sign of population expansion or reduction was detected when regions were considered independently (Figure 4).

### 3.4. ptDNA Dating and Phylogenetic Analysis

The phylogenetic tree resulting from the Bayesian analysis with MrBayes shows how the haplotypes of *D. caudatum* form a monophyletic group, and haplotypes from H-I to H-VII form a strongly supported clade (Figure S4). Within this clade, relationships between haplotypes were not resolved.

**Figure 5:**
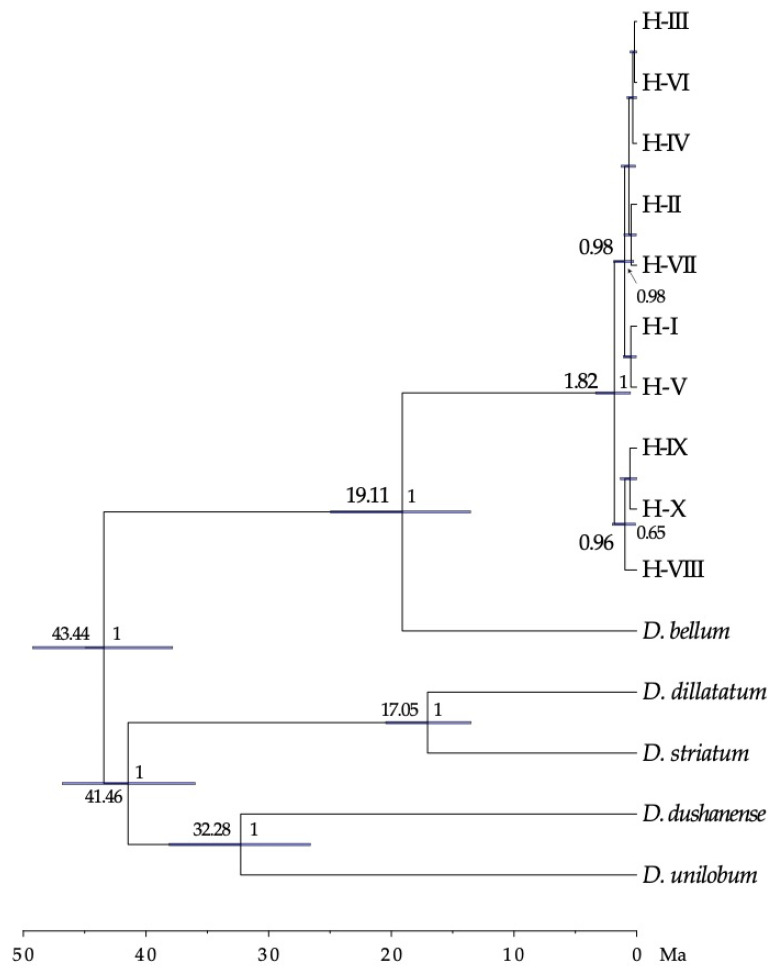
Time-calibrated phylogeny for the *D. caudatum* ptDNA haplotypes, and outgroup species, derived from BEAST. Numbers above branches are the mean divergence ages (in million years ago) for each node; blue bars represent 95% highest posterior density intervals for each node; numbers after nodes are BEAST posterior probabilities; the time scale is printed in million years ago (Ma). Only data for nodes with pp > 0.6 are shown.

The lineage of *D. caudatum*, according to the dating made with BEAST, originated 19.11 [13.54–24.96] million years ago (Ma). The estimated time for the event of initial divergence of the haplotypes of the *trn*S-*trn*G region was 1.82 [0.51–3.35] Ma coinciding with the end of the first interglacial period and beginning of the second Pleistocene glaciation (Figure 5).

### 3.5. Species Distribution Modelling

**Figure 6:**
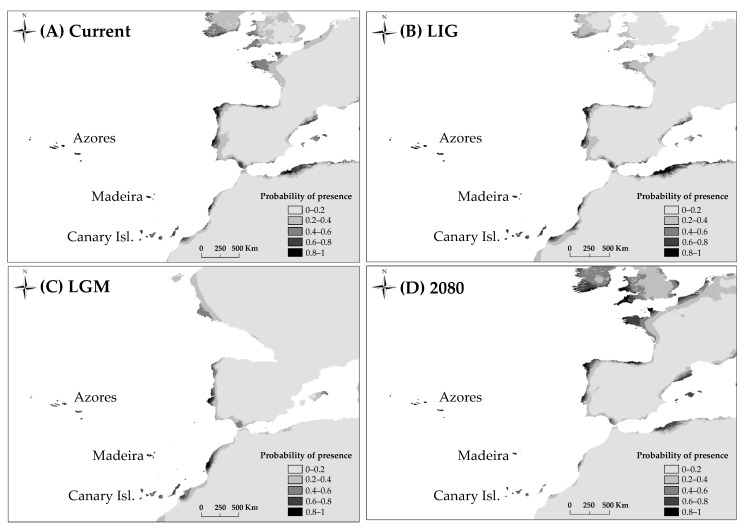
Potential distribution of *D. caudatum* drawn with MAXENT. (**A**), at the present time; (**B**), at the Last Interglacial (LIG, ca. 120,000 years BP); (**C**), at the Last Glacial Maximum (LGM, ca. 21,000 years BP) using the Community Climate System Model (CCSM); (**D**), at 2080 under RCP 8.5 conditions.

For all models AUC values were high (minimum value of AUC = 0.992). The predictions obtained by MAXENT for the present and the LIG show similar suitable areas for the habitat of *D. caudatum* in relation to the current distribution of the species, adding the Portuguese coast as a potential area (Figure 6). According to the LGM outputs, the shelters of this species were located in the Macaronesian islands, the lower half of the Atlantic coast of the Iberian Peninsula and the coast of Morocco. The future projections showed a decrease of the suitability in Andalusia, staying in the Macaronesian archipelagos with a high probability (Figure 6).

## 4. Discussion

### 4.1. Reproductive System Prevalent in D. caudatum

Among ferns, asexuality is considered a common phenomenon where it has been documented in approximately 10% of species [100]. Asexuality can affect the quantity and distribution of genetic diversity, although different extents of clonality will have varying consequences [48,101].

The results we have obtained for the clonal descriptors and AMOVA analyses (with the highest proportion of diversity always found within samples) showed that in *D. caudatum* an outcrossing breeding system predominates (Table 2 and Table 4). In Andalusia, where only 11 clonal lineages were detected between the 35 ramets studied, the low genotypic diversity and evenness values and the significantly high linkage disequilibrium among loci (Table 2), and the high inbreeding coefficient (Table 3), point to a shift in mating system toward increased inbreeding and/or clonality. In line with the predominant mating system, the levels of clonality detected for the whole species did not affect the levels of genetic diversity observed nor the structuring of this diversity. Only for the Andalusian populations, genetic indices varied substantially (Table 3 and Table S2).

In ferns, the reproductive system is closely related to the colonizing capacity of the species. The colonization of new habitats will be more likely for those species with intra-gametophytic selfing, which are able to establish a new population even from just one spore [39,44]. In herbaceous plants with clonal reproduction, after an initial founder event by one or few genotypes, the expansion process can occur via vegetative reproduction (e.g., [102,103]). This form of colonization results in very genetically impoverished populations, especially for outcrossing diploids like *D. caudatum*, since intra-gametophytic selfing generates totally homozygous sporophytes (from the same haploid gametophyte). In order for these populations to increase their genetic diversity and viability sexual reproduction with immigrant genotypes is necessary (something that is highly dependent on the degree of isolation of the population and the time elapsed since its foundation; [104], or that polyploidy events occur which favour the storage of genetic variation via fixed heterozygosity [50,105]. Although we do not have chromosomal data for the populations studied, only those provided by [51] and by [52] for some Madeira and Azores populations respectively, we never found more than two alleles in all individuals genotyped with the microsatellite loci, supporting the diploid nature of the species including the Andalusian populations. These continental populations, with very high homozygosity and very few clonal lineages (contrary to the outcrossing Macaronesian populations), could be examples of colonization from only one or few spores and establishment through selfing and vegetative reproduction. Unlike what happens in the Macaronesian populations, where founder events must also have occurred for the colonization of the islands, the strong isolation of the Andalusian populations (evidenced in the genetic structure analyses; Figure 2 and Figure 3) and/or their relatively recent origin, might be responsible for these populations not having reached high levels of diversity. Ref. [47] showed that intraspecific variation in a mating system may be common and that the genotypes with highest selfing capacity were those in isolated populations, supporting the idea that selection for selfing genotypes may occur during long-distance colonization. The high homozygosity observed in the Andalusian populations of *D. caudatum* with respect to those in Macaronesia suggests a differential capacity for selfing, supporting the idea raised by [47]. Similar results have been found by us in *Culcita macrocarpa*, in which the highly homozygous populations from Andalusia and several from the Cantabrian Cornice were proposed to have resulted from dispersive events from source populations in Azores and others in the Cantabrian Cornice [28].

### 4.2. Tertiary Origin of D. caudatum

Recent biogeographical studies of the genus *Diplazium* place its origin in Asia and the beginning of its diversification at 41.7 Ma. These data are consistent with the idea that *Diplazium* species were part of the Palaeotropical flora [20]. Our dating analysis places *D. caudatum* as part of this palaeoflora during the Miocene (origin of the lineage 19 Ma; Figure 5). MAXENT analysis results showed a current and past potential distribution of *D. caudatum* in the Iberian Peninsula and North Africa much greater than the actual distribution of the species, suggesting that the species has undergone an area reduction over time. The almost total absence of *D. caudatum* in continental Europe (being present only in Andalusia) is probably due to the progressive retraction towards the south and subsequent disappearance of the lauroid-type palaeoflora, as a result of the strong climatic changes from the Middle Miocene onwards [1,15,53]. The Iberian Peninsula is considered the last refuge in Europe of this flora, which persisted until the Late Pliocene (3.5–2.6 Ma; [106]), time when the Mediterranean climate was established (3.2 Ma; [107]). According to [18] *D. caudatum* could be one of the species that survived in Iberian and Macaronesian shelters, like other Tertiary species of ferns. *Diplazium caudatum* joins the list of ferns present in the Macaronesia and Iberian Peninsula for which a Tertiary origin is corroborated, and therefore its relictual nature (*Davallia canariensis*, [26]; *Culcita macrocarpa*, *Vandenboschia speciosa*, [28]). However, as we will see below, our results suggest the disappearance of *D. caudatum* from the mainland and a subsequent back-colonization from Macaronesia.

### 4.3. Genetic Diversity Pattern and Evolutionary History of D. caudatum

Dating results showed that the first haplotype divergence event corresponds to the private haplotypes of Madeira (1.8 Ma; Figure 5). In accordance with the network, these haplotypes are derived from H-III, central and most frequent, so they have likely originated in situ. Therefore, we can suggest the end of the first interglacial as a minimum date for the presence of the species in Macaronesia. However, we might suppose the arrival before glaciations, during the Pliocene, in line with the fossil material from the end of this time found at the deposit of São Jorge in Madeira (spermatophyte and fern species of the Tertiary that occupied the same niche as *D. caudatum*; [108]). According to [19] the arrival of the lauroid forests to the south of the Iberian Peninsula, and with them *D. caudatum*, could occur during the Pliocene, a period where fossils of trees of this palaeoflora, such as *Laurophyllum*, have been recorded and when the region presented available habitats for the persistence of these long-term forests, refugees from the newly established Mediterranean climate [5].

The arrival of *D. caudatum* to the Macaronesia region could be from populations in the Iberian Peninsula. However, our data does not support currently existing populations as source populations of those in Macaronesia. Populations that have persisted for a long time in shelters are expected to harbour high levels of genetic diversity with respect to populations resulting from new colonization or recolonization processes (subject to founder effects and strong genetic drift; see [109]). In island populations the subset of genetic variation of the source population is rapidly fixed as a result of the strong impact of genetic drift and limited gene flow to islands [110]. Contrary to expectations, Andalusian populations showed very low levels of diversity in relation to Macaronesian populations. In addition, the only ptDNA haplotype detected in Andalusia (H-I) was derived from H-III, the most frequent and ancestral haplotype and which is not in Andalusia (Figure 1A,B). Therefore, although unknown and/or recently extinct source Iberian populations are possible, our data are consistent with the fact that the presence of *D. caudatum* in Andalusia is the result of a process of recolonization from Macaronesia, as has been shown for other species not only of angiosperms but also of bryophytes and pteridophytes [10,28,36,111,112,113]. The relationship of COQ with the Canarian populations resulting from genetic structure analysis suggests the Canary Islands as a source region of recolonization (Figure 2 and Figure 3). The CRM population, with only two clonal lineages, seems to be the result of dispersion from COQ.

The fact that Andalusian populations are due to recolonization processes implies that *D. caudatum* disappeared from the continent at some point. This assumption would be consistent with the large temporal gap detected between the estimated date of origin of the lineage of *D. caudatum* (19.11 Ma) and that of initial divergence of haplotypes (1.8 Ma; Figure 5); so that extinction on the continent would have made the most ancient haplotypes disappear. The results of the MAXENT analysis show the disjunct distribution that *D. caudatum* already had during the interglacial period, supporting the negative effect of the increase in aridity on the distribution of the species (Figure 6). Thus, it could be possible that its disappearance occurred during the Late Pliocene, when the Mediterranean climate was established and it is believed that the lauroid-type palaeoflora of the Iberian Peninsula disappeared (3.5–2.6 Ma; [106]), or during the interglacial periods of the Quaternary.

Considering these factors, Macaronesian archipelagos are a reservoir of diversity for *D. caudatum* and have served as a refugium, at least, during the Quaternary glaciations. Our phylogeographic analyses in *Diplazium caudatum*, together with those in *C. macrocarpa* and *V. speciosa* [28] have allowed verification that these fern species fit to the Engler refugium model. These studies, together with those in bryophyte species with similar hyper-Atlantic distribution pattern (Macaronesia and western fringe of Europe), reinforce the idea of Macaronesia as a refuge for spore-producing plants and a source region for back-colonization of Europe [10,113].

The absence of a gradient of genetic diversity through Macaronesia disproves that colonization had occurred as a unidirectional wave (from more diverse to less diverse areas) and supports the homogenising effect of long-distance dispersal, decreasing diversity among populations and regions, as shown by the lack of existence of isolation by distance when Andalusian populations where removed from analysis, and that the differentiation between the Azores and the Canary Islands is less than the differentiation between the Canary Islands and Madeira, despite the fact that the latter two archipelagos are geographically closer (Figure 2 and Figure 3; Tables S2 and S3). The high dispersive capacity of *D. caudatum* is evidenced by the widespread distribution of ancestral haplotypes since both H-III and H-II are equally shared between all the archipelagos (Figure 1A,B). This scenario correlates with recurrent inter-island and inter-archipelago colonization, supporting substantial gene flow. In addition, haplotype H-V, very common in the Canary Islands (IJU and PIJ; Figure 1A) and from which the private haplotype H-IV (in IJU) was derived, is also represented in Azores, suggesting a long-distance dispersal from the Canary Islands to the Azores. The inconclusive results of the demographic analyses could be reflecting this recurrent gene flow model. While the Fu’s *F* and Tajima’s *D* analyses supported Madeira and the Azores as regions from where *D. caudatum* expanded its distribution range (Table S5), the BSP supported a general species expansion, but not from any specific region (Figure 4). Recently, the “surfing syngameon hypothesis” has been proposed to explain the absence of a diversity gradient in the colonization processes of the Macaronesian island systems [112]. This hypothesis argues that secondary contacts and subsequent gene flow in island habitats between genotypes that may have previously been isolated on the continent or other island regions generated syngameons that enhanced genetic diversity. This hypothesis would explain similar levels of genetic diversity in *D. caudatum* among the three involved archipelagos.

While the ancestral haplotypes of *D. caudatum* are widely dispersed between islands and archipelagos, the rest (and most recent haplotypes) are private to single islands (Figure 1A, Table 3). In addition, we have also found a high proportion of private alleles to single islands in the microsatellite loci. The widespread distribution of ancestral haplotypes could involve early dispersal after *D. caudatum* initially colonized Macaronesia; while the recent-most haplotypes and microsatellite private alleles suggest a relatively recent lineage-diversification process. This pattern has been found in other Macaronesian plants, as *Picconia azorica* [114] and *Juniperus brevifolia* [115], and it is the first evidence for ferns. [115] proposed the habitat competition with early lineages to explain the pattern observed in *J. brevifolia*. In *D. caudatum*, given the exclusivity of its habitat, this hypothesis would also be possible. This could be especially true during interglacial/postglacial periods, given the negative impact that increased aridity had on the proportion of suitable habitat (Figure 6). Arid periods would have reduced the number of populations and extent of the species, affecting connectivity (decreasing it) between populations within the same archipelago and favouring the isolation and diversification of lineages, and making it harder for immigrants to settle in previously colonized habitats.

### 4.4. Implications for Conservation

According to the SDM for 2080, under the current greenhouse gas emission conditions (RCP 8.5; [116]), the distribution range of the species will be reduced, especially in Andalusia and the Canary Islands (Figure 6). *Diplazium caudatum* is a Tertiary relict and therefore, the conservation of the species and habitats where it is found is of great importance. Andalusian populations, given their pioneering nature in a phase of recolonization and the low diversity they host, deserve special consideration for their protection. With respect to Madeira, this area hosts great diversity, and it would be desirable to give it special consideration for biodiversity conservation and management.

## Figures and Tables

**Table 1:** Sampling details of *D. caudatum* populations used in the present study.

Code	Location	Voucher	Geographical Coordinates	Sample Size	
				Microsatellites	ptDNA
Andalusia					
COQ	Cádiz: Canuto de Ojén Quesada	GDA 65379	N36.127°/W5.585°	17	5
CRM	Cádiz: Cabecera del río de la Miel	GDA 65378	N36.105°/W5.528°	18	5
Azores					
AGU	Terceira: Agual	GDA 65382	N32.734°/W16.886°	30	5
CID	São Miguel: Sete Cidades	GDA 65383	N37.835°/W25.788°	30	5
FUR	São Miguel: Furnas	GDA 65381	N28.134°/W17.273°	30	5
SER	Terceira: Serreta	GDA 65380	N28.119°/W17.224°	30	5
Canary Isl.					
ANC	La Gomera: Ancón Negro	GDA65386	N28.134°/W17.273°	30	5
CED	La Gomera: Cedro	GDA 65389	N28.120°/W17.225°	30	5
IJU	Tenerife: Ijuana	GDA 65388	N28.560°/W16.172°	30	5
PIJ	Tenerife: El Pijaral	GDA 65387	N28.553°/W16.188°	30	5
Madeira					
FRI	Ribeiro Frio	GDA 65384	N32.734°/W16.886°	30	5
POR	Levada Portela	GDA 65385	N32.747°/W16.823°	30	5

**Table 2:** Clonality descriptors in the populations of *D. caudatum* studied. Descriptors were separated into clonal richness, genotype diversity, and linkage disequilibrium. N, number of individuals sampled; MLL, number of different multilocus lineages; eMLL, number of expected MLLs at the smallest sample size ≥ 17 based on rarefaction [92]; R, clonal richness [93]; lambda, Simpson’s index [94]; E.5, evenness [95,96,97]; *r*_d_, standardized index of association [60]; *r*_d_ wc, *r*_d_ calculated considering only one individual per MLL per population.

Population	*N*	Clonal Richness			Genotype Diversity		Linkeage Disequilibrium	
		MLL	eMLL	R	Lambda	E.5	*r*_d_	*r*_d_ wc
Andalusia	35	11	11	0.294	0.798	0.587	0.334 *	0.103 *
COQ	17	9	9	0.500	0.912	0.875	0.107 *	0.047
CRM	18	2	2	0.059	0.294	0.676	NA	NA
Azores	120	107	33.3	0.883	0.996	0.845	0.015	−0.002
AGU	30	30	17	1	1	1	−0.018	−0.018
CID	30	28	16.23	0.931	0.993	0.927	−0.019	−0.028
FUR	30	26	15.6	0.862	0.989	0.915	0.080 *	0.054 *
SER	30	26	15.6	0.862	0.989	0.915	0.047	0.016
Canary Isl.	120	114	34.44	0.949	0.999	0.964	0.021 *	0.019 *
ANC	30	29	16.7	0.965	0.998	0.981	0.019	0.014
CED	30	27	15.9	0.896	0.991	0.92	0.112 *	0.088 *
IJU	30	29	16.7	0.965	0.998	0.981	0.007	0.002
PIJ	30	29	16.7	0.965	0.998	0.981	0.017	0.005
Madeira	60	59	34.66	0.983	0.999	0.990	0.007	0.003
FRI	30	30	17	1	1	1	0.018	0.018
POR	30	29	16.7	0.965	0.998	0.981	0.022	0.008
Total	335	290	16.67	0.865	0.997	0.682	0.104	0.099

* *p* < 0.05; NA, not applicable; wc, without clones.

**Table 3:** Genetic diversity indices for microsatellites and ptDNA sequences in the populations of *D. caudatum* studied. Indices were calculated including all individuals sampled per population and including only one individual per MLLs per population (wc; without clones). N, number of individuals sampled; *A*, number of alleles with unique alleles in brackets; *Ar*, allelic richness at the smallest sample size (34 and 4 for populations with clones and without clones, respectively; 70 and 20 for geographical regions with clones and without clones, respectively) based on rarefaction; *H*_O_, observed heterozygosity; *H*_E_, expected heterozygosity [98]; *F*_IS_, inbreeding coefficient [99]; *ha*: number of haplotypes with unique haplotypes in brackets; *Hd*: haplotype diversity; *π*: nucleotide diversity.

Population	N	Microsatellites									ptDNA		
		*A* (Private)	*Ar*	*Ar* wc	*H*_O_	*H*_O_ wc	*H*_E_	*H*_E_ wc	*F*_IS_	*F*_IS_ wc	*ha* (Private)	*Hd*	*π* (×10^3^)
Andalusia	35	13	1.625	1.625	0.025	0.045	0.228	0.297	0.890 *	0.847 *	1 (1)	0.000	0.00
COQ	17	13	1.625	1.478	0.029	0.042	0.251	0.283	0.883 *	0.853 *	1	0.000	0.00
CRM	18	9	1.125	1.125	0.021	0.063	0.020	0.063	−0.063	0.000	1	0.000	0.00
Azores	120	42 (4)	4.203	3.179	0.237	0.251	0.353	0.369	0.328 *	0.319 *	5 (2)	0.663	1.14
AGU	30	23	2.757	1.748	0.263	0.263	0.357	0.357	0.264 *	0.264 *	2	0.600	0.83
CID	30	25 (1)	2.871	1.722	0.321	0.335	0.334	0.348	0.041	0.037	2	0.600	0.83
FUR	30	25 (2)	2.874	1.723	0.171	0.178	0.328	0.347	0.479 *	0.488 *	4 (1)	0.900	1.66
SER	30	29 (1)	3.202	1.722	0.196	0.221	0.314	0.342	0.377 *	0.353 *	3 (1)	0.800	1.39
Canary Isl.	120	48 (4)	4.930	3.685	0.224	0.230	0.428	0.428	0.476 *	0.463 *	4 (1)	0.679	1.41
ANC	30	22	2.586	1.661	0.200	0.198	0.316	0.318	0.367 *	0.377 *	1	0.000	0.00
CED	30	23 (1)	2.679	1.712	0.183	0.204	0.340	0.335	0.460 *	0.392 *	2	0.600	0.83
IJU	30	24 (1)	2.840	1.742	0.233	0.233	0.340	0.343	0.314 *	0.322 *	2 (1)	0.900	1.94
PIJ	30	31 (2)	3.441	1.826	0.279	0.284	0.381	0.386	0.267 *	0.263 *	4	0.600	1.66
Madeira	60	86 (37)	9.576	6.514	0.417	0.417	0.630	0.630	0.338 *	0.338 *	5 (3)	0.822	1.82
FRI	30	69 (11)	7.282	2.567	0.438	0.438	0.626	0.626	0.301 *	0.301 *	3 (1)	0.700	1.94
POR	30	66 (12)	6.915	2.535	0.396	0.397	0.615	0.617	0.356 *	0.357 *	4 (1)	0.900	1.94
Total	335	45			0.227	0.238	0.495	0.505	0.354 *	0.362 *	10 (7)	0.7836	1.76

* *p* < 0.05; wc, without clones.

**Table 4:** Hierarchical analysis of molecular variance (AMOVA).

Souce of Variation	d.f.	Sum of Squares	Percentage of Variation	Phi	*p*-Value
***Microsatellites***					
**All sampling units**					
*Standard*					
Within samples	335	650	48.19	0.518	<0.001
Between samples within populations	323	1274.937	24.92	0.341	<0.001
Between populations	11	706.699	26.89	0.269	<0.001
Total	669	2631.635	100		
*Hierarchical* (4 geographical regions)					
Within samples	335	650	46.37	0.536	<0.001
Between samples within populations	323	1274.937	23.98	0.341	<0.001
Between populations within regions	8	288.806	13.52	0.161	<0.001
Between regions	3	417.793	16.13	0.161	<0.001
Total	669	2631.636	100		
**MLLs**					
*Standard*					
Within samples	294	632	53.75	0.462	<0.001
Between samples within populations	282	1194.834	26.09	0.327	<0.001
Between populations	11	476.138	20.16	0.201	<0.001
Total	587	2302.973	100		
*Hierarchical* (4 geographical regions)					
Within samples	294	632	52.28	0.477	<0.001
Between samples within populations	282	1194.834	25.38	0.327	<0.001
Between population within regions	8	230.767	11.93	0.133	<0.001
Between regions	3	245.372	10.41	0.104	0.002
Total	587	2302.973	100		
***ptDNA***					
*Hierarchical* (4 geographical regions)					
Within populations	48	13.200	64.41	0.356	<0.001
Between populations within regions	8	3.250	6.15	0.087	0.161
Between regions	3	6.667	29.44	0.294	0.007
Total	52	23.117	100		

## Data Availability

All sequence data obtained in this study, ptDNA and microsatellites, are available in the GenBank database (ptDNA accession numbers: OL311491-OL311500; microsatellite accession numbers: OL311506-OL311513).

## Supplementary Materials

The following are available online at https://www.mdpi.com/article/10.3390/plants10112425/s1, Figure S1: Genotypic accumulation curve showing the resolutive power of the eight microsatellite used in this study, Figure S2: Histogram of frequency distribution of pairwise genetic distances, Figure S3: Distribution of the 290 MLLs among the 294 individuals (genets) of *D. caudatum*, Figure S4: 50% majority-rule consensus tree obtained from the Bayesian inference with MrBAYES of the *trn*S-*trn*G ptDNA region of *D.caudatum* and outgroup species, Table S1: Characteristics of 8 developed microsatellite loci, Table S2: Percent contribution and permutation importance (MaxEnt) of selected model, Table S3: Pairwise population *F*_ST_ for microsatellites, Table S4: Mean recent migration rates (*m*) among the studied populations, estimated from eight microsatellite loci using the BAYESASS program, Table S5: Neutrality tests Fu’s *F* and Tajima’s *D* of the populations and regional groups of *D. caudatum* performed to measure population expansion and contraction.
