# Supplementary material for: Macaronesia Acts as a Museum of Genetic Diversity of Relict Ferns: The Case of Diplazium caudatum (Athyriaceae)
Source: Plants (Basel). 2021 Nov 10;10(11):2425. doi: 10.3390/plants10112425 (PMC8623695; doi:10.3390/plants10112425)
Supplement: Supplementary file 1 [file plants-10-02425-s001.zip › plants-1419944-supplementary.pdf]

Supporting information

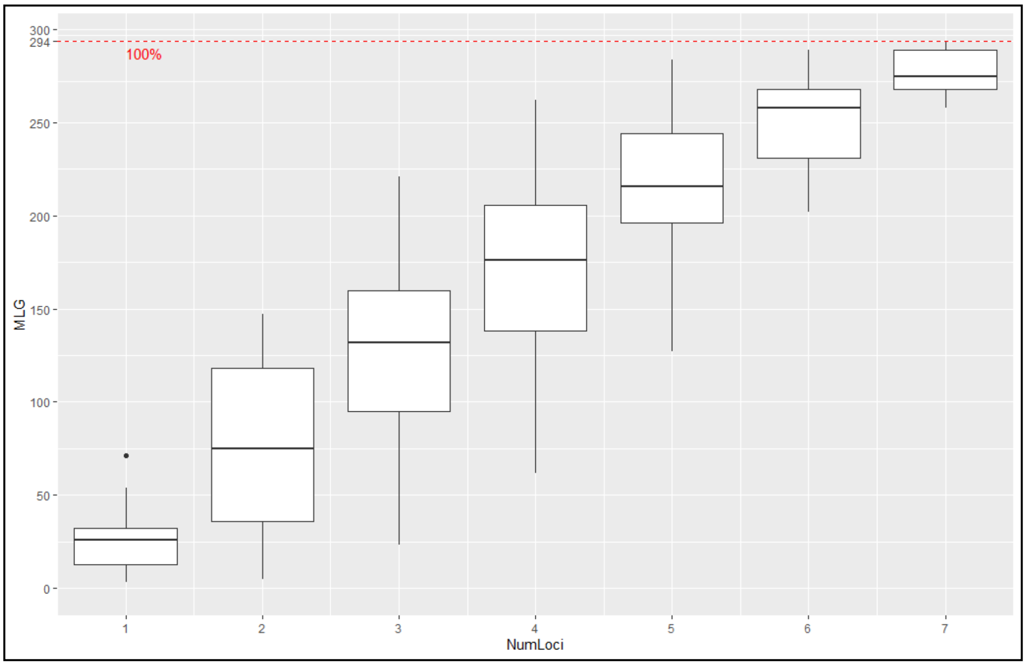

Figure S1. Genotypic accumulation curve showing the resolutive power of the eight microsatellite used in this study.

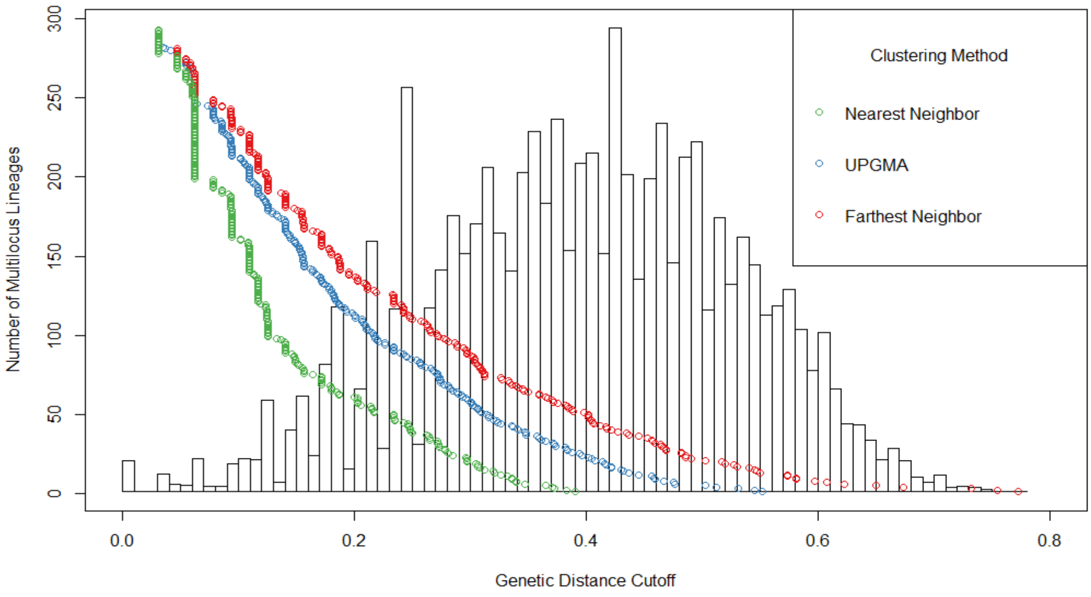

Figure S2. Histogram of frequency distribution of pairwise genetic distances. The genetic threshold distances (for the three methods implemented in POPPR) under which two MLGs are considered the same MLL are showed.

a)

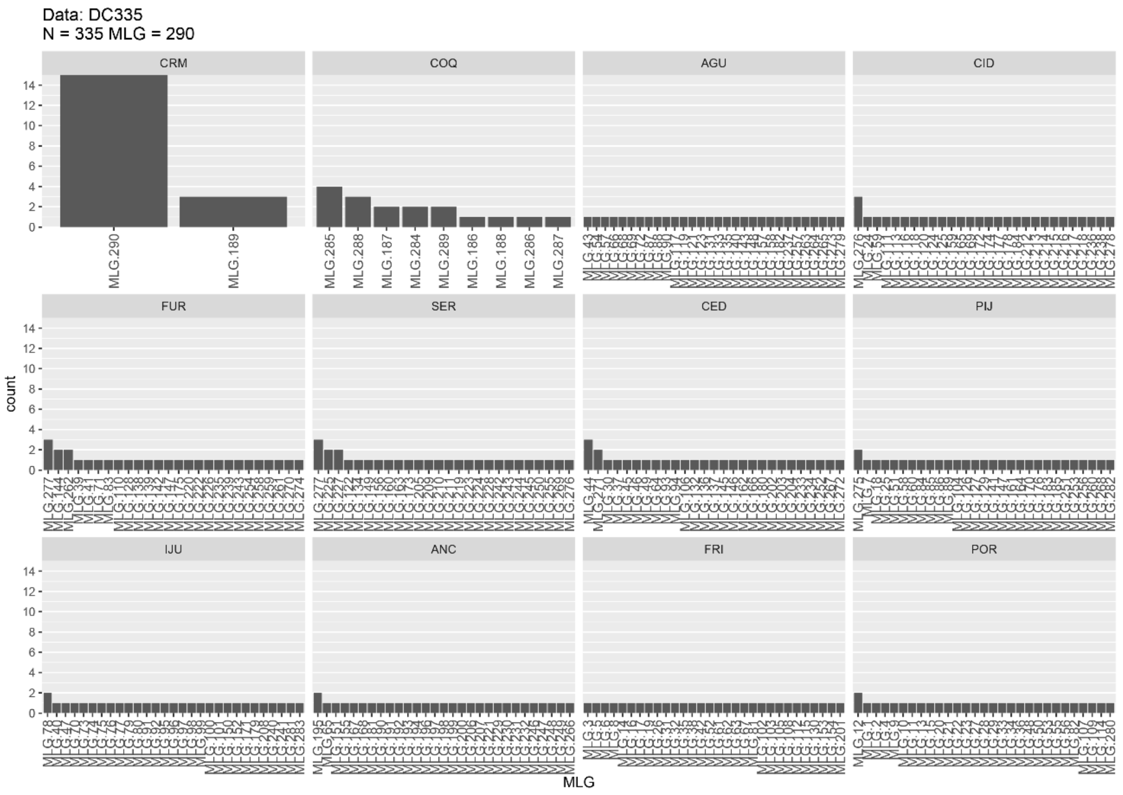

b)

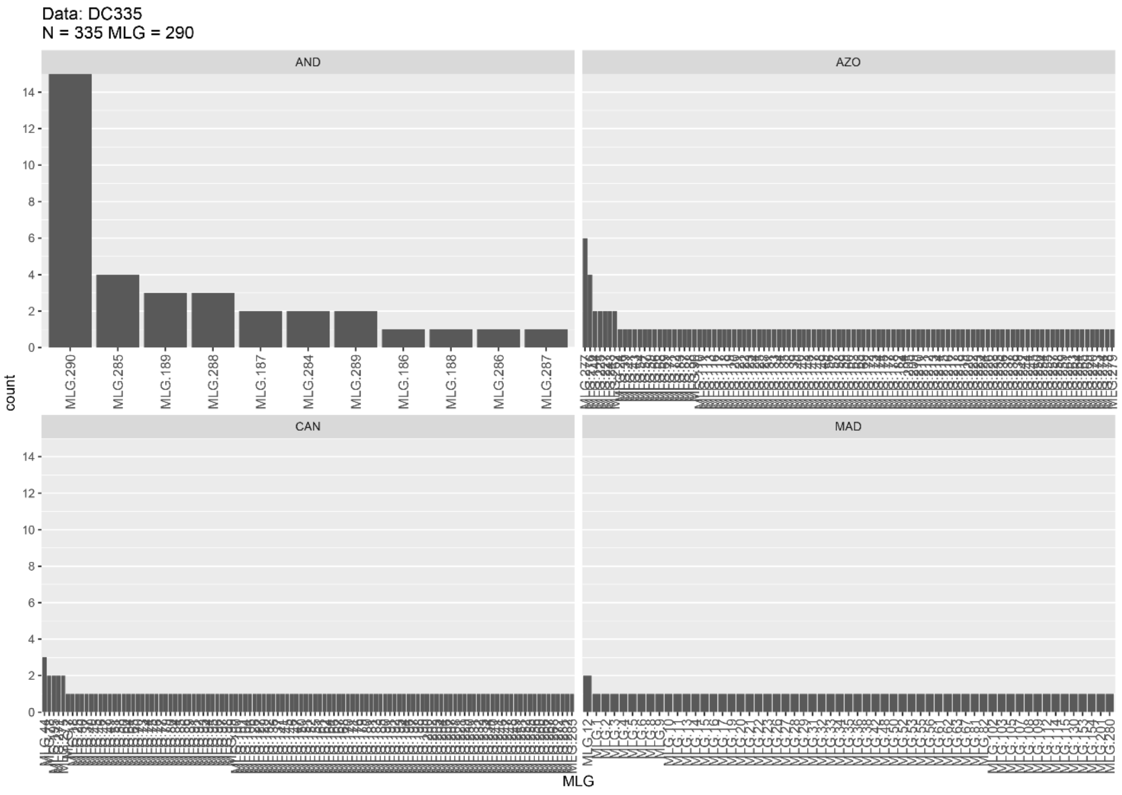

Figure S3. Distribution of the 290 MLLs among the 294 individuals (genets) of *D. caudatum*. Across the 12 populations (a) and across the 4 geographical regions (b).

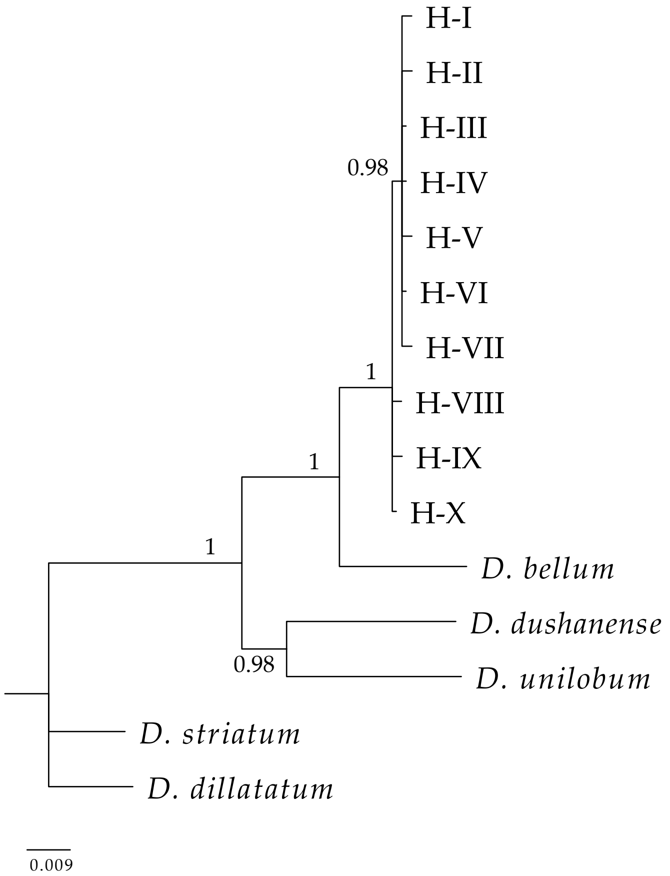

Figure S4. 50% majority-rule consensus tree obtained from the Bayesian inference with MrBAYES of the *trn*S-*trn*G ptDNA region of *D.caudatum* and outgroup species. Haplotypes are denoted with Roman numerals; numbers above branches are Bayesian posterior probabilities.

Table S1. Characteristics of 8 developed microsatellite loci.

Locus	Primer sequences (5′–3′)[a]	Repeat motif	Allele size range (bp)	T_a (°C)[b]	GenBank accession no.
DC-27	F: AGCAGAGGAGCAGTGGAA<br>R: TCTTGAGCGAGTAGAGGTAA	(GA)23	172-192	52	OL311506
DC-66A	F: TCTCGCAAACAATCAGTAAA<br>R: GGTTGTTGTTGCAGGAAAT	[A(C/G)]21	252	52	OL311507
DC-77	F: CTAACTGAGCGAGATAAG<br>R: CGCGAAACAGGGCAAGAG	(AG)25	184-250	52	OL311508
DC-AT12	F: CATCTTTTCATGGGTACAAG<br>R: GGTAGATCACTGCACATTG	(CT)15	156-170	52	OL311510
DC-AT17	F: AAAAGCCTAGATTTCTCAT<br>R: TTATGCAAATCAGGTTTAT	(GA)18	207-285	48	OL311511
DC-AT30	F: TCGCCCGATGTTGTGTAATT<br>R: GTTTACGAACCTGTAGCCATCAGT	(GT)12	320-324	58	OL311513
DC-AT2	F: TGTCCCTTATCATTGCACAGG<br>R: GGACTACTCCCACGATGTCA	(GA)22	148-182	60	OL311509
DC-AT22	F: AGAGGGTGAGTCAGTGGA<br>R: ATATGGTGATTTAGATAGGCCAG	(GA)23	286-328	58	OL311512

Note: T_a = annealing temperature.

[a] Forward primer sequence and allele size range do not include the M13-tail sequence (5′-CACGACGTTGTAAAACGAC-3′).

[b] Cycling parameters: 94°C 3min, followed by 35 cycles of 94°C 30 s, T_a 30 s, and 72°C 30 s; and a final extension at 72°C 10 min. PCR reactions: 10-μL containing 20 ng of genomic DNA, 0.02 μM of the M13-labeled forward primer, 0.45 μM of each reverse primer and fluorolabeled M13 primer (5′-6FAM, 5′-HEX, 5′-ATTO-550), 5 μL Kapa 2G Robust HotStart ReadyMix (Kapa Biosystems, Boston, MA).

Table S2. Percent contribution and permutation importance (MaxEnt) of selected model. Variables in bold were selected for the final model.

Variable	MaxEnt Percent contribution	MaxEnt Permutation importance
**Mean Diurnal Range**	**52.5**	**26.2**
**Min. Temperature of Coldest Month**	**24.1**	**70.3**
Precipitation of Warmest Quarter	16.7	3.2
Type of Soil	2.5	0.1
Precipitation of Driest Month	1.9	0.0
Precipitation Seasonality	1.8	0.0
Precipitation of Coldest Quarter	0.3	0.1
Max. Temperature of Warmest Month	0.2	0.1
Precipitation of Wettest Month	0.1	0.0

Table S3. Pairwise population F_{ST} for microsatellites. F_{ST} values obtained with all sampled individuals are represented below the principal diagonal, and those obtained with a single representing MLL per population above the principal diagonal.Values in bold were significant at the 5% nominal level after sequential Bonferroni correction.

	**CRM**	**COQ**	**AGU**	**CID**	**FUR**	**SER**	**CED**	**PIJ**	**IJU**	**ANC**	**FRI**	**POR**
**CRM**	**--**	0.306	0.504	0.544	0.540	0.503	0.566	0.481	0.566	0.506	0.317	0.310
**COQ**	**0.586**	**--**	**0.295**	**0.328**	**0.338**	**0.283**	**0.373**	**0.304**	**0.350**	**0.378**	**0.284**	**0.314**
**AGU**	**0.631**	**0.315**	**--**	**0.086**	**0.075**	**0.081**	**0.111**	**0.058**	**0.163**	**0.263**	**0.166**	**0.224**
**CID**	**0.667**	**0.353**	**0.089**	**--**	**0.056**	**0.072**	**0.123**	**0.110**	**0.194**	**0.292**	**0.183**	**0.237**
**FUR**	**0.675**	**0.364**	**0.071**	**0.057**	**--**	0.049	**0.099**	**0.082**	**0.188**	**0.273**	**0.176**	**0.228**
**SER**	**0.653**	**0.314**	**0.088**	**0.082**	0.058	**--**	**0.121**	**0.123**	**0.197**	**0.225**	**0.199**	**0.246**
**CED**	**0.674**	**0.385**	**0.102**	**0.126**	**0.098**	**0.123**	**--**	**0.142**	**0.180**	**0.336**	**0.178**	**0.244**
**PIJ**	**0.616**	**0.325**	**0.057**	**0.108**	**0.075**	**0.131**	**0.138**	**--**	**0.170**	**0.247**	**0.156**	**0.199**
**IJU**	**0.685**	**0.374**	**0.169**	**0.209**	**0.204**	**0.220**	**0.178**	**0.180**	**--**	**0.330**	**0.175**	**0.223**
**ANC**	**0.635**	**0.399**	**0.268**	**0.301**	**0.279**	**0.240**	**0.334**	**0.254**	**0.341**	**--**	**0.266**	**0.288**
**FRI**	**0.478**	**0.317**	**0.166**	**0.195**	**0.192**	**0.222**	**0.175**	**0.160**	**0.179**	**0.270**	**--**	**0.027**
**POR**	**0.477**	**0.349**	**0.225**	**0.251**	**0.248**	**0.273**	**0.244**	**0.206**	**0.228**	**0.287**	**0.029**	**--**

Table S4. Mean recent migration rates (*m*) among the studied populations, estimated from eight microsatellite loci using the BAYESASS program. Values on the diagonal (underlined) indicate the proportion of individuals in each generation that are not migrants. Values in bold are the *m* rates that are informative.

From<br>To	CRM	COG	AGU	CID	FUR	SER	CED	PIJ	IJU	ANC	FRI	POR
**CRM**	0.689<br>(0.021)	0.085<br>(0.037)	0.023<br>(0.021)	0.021<br>(0.020)	0.022<br>(0.021)	0.022<br>(0.020)	0.024<br>(0.024)	0.021<br>(0.019)	0.022<br>(0.021)	0.022<br>(0.021)	0.022<br>(0.021)	0.022<br>(0.02)
**COQ**	0.016<br>(0.016)	0.791<br>(0.049)	0.037<br>(0.027)	0.015<br>(0.014)	0.016<br>(0.016)	0.014<br>(0.014)	0.028<br>(0.038)	0.016<br>(0.015)	0.015<br>(0.015)	0.016<br>(0.016)	0.016<br>(0.015)	0.015<br>(0.014)
**AGU**	0.008<br>(0.007)	0.008<br>(0.008)	0.909<br>(0.023)	0.008<br>(0.008)	0.007<br>(0.007)	0.008<br>(0.007)	0.010<br>(0.010)	0.007<br>(0.007)	0.008<br>(0.008)	0.008<br>(0.007)	0.008<br>(0.008)	0.008<br>(0.007)
**CID**	0.008<br>(0.008)	0.008<br>(0.008)	**0.243**<br>(0.022)	0.674<br>(0.008)	0.008<br>(0.008)	0.008<br>(0.007)	0.008<br>(0.007)	0.007<br>(0.007)	0.008<br>(0.008)	0.008<br>(0.007)	0.008<br>(0.007)	0.008<br>(0.007)
**FUR**	0.009<br>(0.008)	0.009<br>(0.009)	**0.224**<br>(0.025)	0.008<br>(0.008)	0.675<br>(0.008)	0.008<br>(0.008)	0.013<br>(0.012)	0.009<br>(0.008)	0.008<br>(0.008)	0.015<br>(0.012)	0.008<br>(0.008)	0.008<br>(0.008)
**SER**	0.009<br>(0.009)	0.009<br>(0.008)	**0.236**<br>(0.025)	0.008<br>(0.008)	0.008<br>(0.008)	0.675<br>(0.008)	0.010<br>(0.010)	0.008<br>(0.008)	0.008<br>(0.008)	0.009<br>(0.009)	0.008<br>(0.008)	0.008<br>(0.008)
**CED**	0.009<br>(0.009)	0.009<br>(0.008)	0.079<br>(0.033)	0.008<br>(0.007)	0.008<br>(0.008)	0.008<br>(0.008)	0.835<br>(0.035)	0.008<br>(0.008)	0.008<br>(0.008)	0.008<br>(0.008)	0.008<br>(0.008)	0.008<br>(0.008)
**PIJ**	0.008<br>(0.008)	0.008<br>(0.008)	**0.242**<br>(0.026)	0.007<br>(0.007)	0.007<br>(0.007)	0.007<br>(0.007)	0.008<br>(0.008)	0.677<br>(0.015)	0.009<br>(0.009)	0.008<br>(0.008)	0.007<br>(0.007)	0.008<br>(0.007)
**IJU**	0.008<br>(0.008)	0.008<br>(0.008)	**0.212**<br>(0.057)	0.008<br>(0.008)	0.008<br>(0.008)	0.008<br>(0.008)	0.009<br>(0.008)	0.008<br>(0.008)	0.704<br>(0.052)	0.008<br>(0.008)	0.008<br>(0.008)	0.008<br>(0.007)
**ANC**	0.008<br>(0.007)	0.008<br>(0.008)	0.027<br>(0.015)	0.007<br>(0.007)	0.008<br>(0.008)	0.008<br>(0.008)	0.009<br>(0.008)	0.007<br>(0.007)	0.008<br>(0.008)	0.892<br>(0.024)	0.007<br>(0.007)	0.008<br>(0.008)
**FRI**	0.008<br>(0.008)	0.013<br>(0.012)	0.02<br>(0.015)	0.009<br>(0.009)	0.009<br>(0.009)	0.009<br>(0.009)	0.024<br>(0.019)	0.009<br>(0.009)	0.019<br>(0.020)	0.009<br>(0.009)	0.844<br>(0.040)	0.022<br>(0.021)
**POR**	0.008<br>(0.008)	0.01<br>(0.009)	0.01<br>(0.01)	0.008<br>(0.007)	0.008<br>(0.007)	0.008<br>(0.008)	0.010<br>(0.009)	0.009<br>(0.009)	0.012<br>(0.014)	0.009<br>(0.008)	0.041<br>(0.025)	0.864<br>(0.032)

Table S5. Neutrality tests Fu's *F* and Tajima's *D* of the populations and regional groups of *D. caudatum* performed to measure population expansion and contraction. Values in bold are significant; N.A.: not applicable

**Region/Population**	***F***	***P*-value**	***D***	***P*-value**
Andalusia	0.000	N.A.	0.000	1
CRM	0.000	N.A.	0.000	1
COQ	0.000	N.A.	0.000	1
Azores	-1.625	0.093	-0.393	0.383
AGU	0.626	0.511	1.225	0.937
CID	0.626	0.526	1.225	0.935
FUR	**-1.938**	0.014	-0.972	0.1
SER	-0.474	0.200	0.243	0.769
Canary Islands	0.039	0.514	0.554	0.744
ANC	0.000	N.A.	0.000	1
CED	0.626	0.513	1.225	0.930
PIJ	1.687	0.761	1.459	0.952
IJU	**-1.648**	0.047	-0.175	0.439
Madeira	-1.587	0.057	-0.279	0.381
FRI	0.061	0.320	-0.175	0.432
POR	**-1.648**	0.047	-0.175	0.432
